# Perceived organizational support profiles and their moderating role in the association between emotional labor and work engagement among Chinese healthcare workers: a latent profile analysis

**DOI:** 10.1186/s12913-026-14626-8

**Published:** 2026-05-09

**Authors:** Hongbin Cong, Afei Qin, Xinru Bian, Hongwei Nie, Ligang Xu

**Affiliations:** 1 State Key Laboratory of Reproductive Medicine and Offspring Health, Center for Reproductive Medicine, Institute of Women, Children and Reproductive Health, Shandong University, Jinan, Shandong, 250012 China; 2 Department of Social Medicine and Health Management, School of Public Health, Cheeloo College of Medicine, Shandong University, Jinan, Shandong, 250012 China; 3 NHC Key Lab of Health Economics and Policy Research, (Shandong University), Jinan, Shandong, 250012 China; 4 Center for Health Management and Policy Research, Shandong University (Shandong Provincial Key New Think Tank), Jinan, Shandong, 250012 China; 5 School (Institute) of Mental Health and Psychological Sciences, Cheeloo College of Medicine, Shandong University, Jinan, Shandong, 250012 China; 6 Personnel Department, Shandong University, Jinan, Shandong, 250100 China; 7 Department of Organization and Personnel (Talent Work Office), Shandong Provincial Hospital Affiliated to Shandong First Medical University, No. 324, Jingwuweiqi Road, Huaiyin District, Jinan, Shandong, 250021 China

**Keywords:** Healthcare workforce, Emotional labor, Work engagement, Perceived organizational support, Latent profile analysis

## Abstract

**Background:**

Emotional labor is a common job demand in healthcare and may reduce healthcare workers’ work engagement, potentially affecting workforce sustainability and service quality. Perceived organizational support (POS) may buffer this adverse effect, yet its potential latent heterogeneity is often overlooked. This study aimed to identify latent POS profiles and examine whether these profiles moderate the association between emotional labor and work engagement among Chinese healthcare workers.

**Methods:**

A cross-sectional survey was conducted between August and November 2023 among 1503 healthcare workers from 16 tertiary public hospitals in Shandong Province, China. POS was measured using the 24-item Chinese Employee Perceived Organizational Support Scale, WE was assessed with the Utrecht Work Engagement Scale, and emotional labor was measured using the Emotional Labor Scale. Latent profile analysis (LPA) was used to identify POS profiles. Multivariable ordinary least squares regression models examined associations and moderating effects. Supplementary analyses examined moderation using continuous POS measures and restricted cubic spline models.

**Results:**

Three distinct POS profiles were identified: low (18.1%), medium (52.0%), and high (29.9%). Emotional labor was significantly negatively associated with work engagement (β = −0.35, *p* < 0.001). Compared with the low POS profile, healthcare workers in the medium (β = 10.65, 95% CI: 8.61–12.70, *p* < 0.001) and high (β = 21.74, 95% CI: 19.34–24.14, *p* < 0.001) profiles reported higher work engagement. Moderation analyses showed that the negative association between emotional labor and WE was significantly attenuated in the high POS profile (interaction β = 0.43, *p* < 0.001), whereas the medium POS profile did not show a significant buffering effect (interaction β = 0.11, *p* = 0.226). Findings were consistent when POS was modeled as a continuous variable, and restricted cubic spline analyses suggested that the buffering effect of POS may become more pronounced at higher levels of support.

**Conclusion:**

POS showed clear latent heterogeneity and may function as an important buffering resource in the association between emotional labor and work engagement. Strengthening organizational support systems, particularly for employees with low perceived support, may help sustain work engagement in high-demand healthcare settings.

**Clinical trial number:**

Not applicable.

**Supplementary information:**

The online version contains supplementary material available at 10.1186/s12913-026-14626-8.

## Introduction

Among various job demands, emotional labor, a concept originally developed by Hochschild, has been recognized as one of the most prominent sources of psychological strain in healthcare settings [1, 2]. In medical practice, clinical empathy itself can be regarded as a form of emotional labor. Healthcare workers are required to consciously regulate their internal feelings and external emotional expressions in accordance with organizational and professional display rules in order to convey warmth, patience, and trustworthiness to patients [3, 4]. Such continuous emotional regulation often consumes substantial psychological resources and may lead to emotional exhaustion and reduced motivation. According to the Job Demands–Resources (JD–R) model [5–8], emotional labor represents an important job demand, and excessive accumulation of such demands may deplete individuals’ psychological energy and weaken their positive work engagement. Previous studies have shown that high levels of emotional labor not only increase psychological and physiological burdens among healthcare workers [9], but also reduce job satisfaction [10], increase the risk of job burnout [11], and ultimately affect the quality of healthcare services and patient safety [12]. Within high-intensity healthcare systems, particularly in the Chinese context, healthcare workers in public hospitals often face heavy clinical workloads and high societal expectations, which may further intensify their occupational stress and emotional demands. Therefore, from the perspective of the interaction between job demands and organizational resources, exploring the mechanisms through which emotional labor influences work engagement is of important theoretical and practical significance.

In the fields of organizational behavior and psychology, perceived organizational support (POS) has been widely recognized as a key organizational resource and an important theoretical construct for explaining employees’ work motivation and work engagement. Workplace support may originate from multiple sources, including the organization, supervisors, and colleagues, and these sources collectively shape employees’ overall perceptions of organizational support [13]. Eisenberger and colleagues [14, 15] proposed that when employees perceive emotional and instrumental support from their organization, they are more likely to develop a stronger sense of responsibility and organizational belonging, which in turn promotes positive work engagement. Work engagement refers to a positive, fulfilling work-related state characterized by vigor, dedication, and absorption [16]. According to the JD–R model [5–7], POS functions as a critical job resource that can buffer the negative effects of job demands while fostering positive work states. Existing research also suggests that the effects of POS may vary across different cultural contexts and organizational environments [17]. Therefore, under conditions of high emotional labor demands, POS may play a moderating role in the relationship between emotional labor and work engagement, such that higher levels of POS may alleviate the negative impact of emotional labor on work engagement.

However, existing studies have typically treated POS as a continuous variable, implicitly assuming relative homogeneity in how support is distributed across healthcare workers [18–20]. Although such variable-centered approaches are useful for estimating average associations, they may provide limited insight into whether employees cluster into subgroups with different overall levels or patterns of perceived support. In reality, healthcare workers may differ not only in the degree of support they perceive, but also potentially in dimensions such as work support, identifying value, and caring about well-being. In contrast, person-centered approaches are able to identify subgroups of individuals who share similar characteristics [21]. Among these methods, latent profile analysis (LPA), a commonly used person-centered statistical technique, has increasingly been applied in organizational behavior and occupational health research [22]. This approach allows researchers to explore whether individuals cluster into empirically distinguishable subgroups, which may reflect either differences in overall support level or variation in dimensional response patterns. Recent studies have further demonstrated that employees may form different support profiles in the workplace, and these profiles are closely associated with various work attitudes and behavioral outcomes [23]. Identifying latent profiles of POS may therefore complement variable-centered analyses by helping distinguish employees with comparatively lower or higher organizational support and by examining whether the association between emotional labor and work engagement differs across such subgroups.

Based on this perspective, the present study focuses on healthcare workers in China. First, latent profile analysis was employed to identify latent classes of POS. Second, the moderating role of different POS profiles in the relationship between emotional labor and work engagement was examined. This study aims to identify subgroups of POS among healthcare workers and to examine whether the association between emotional labor and work engagement varies across these subgroups. By incorporating a person-centered approach, the present study seeks to extend existing variable-centered research and to provide further empirical evidence for understanding differences in healthcare workers’ work states under conditions of high emotional labor.

## Methods

### Study design and data source

This study adopted a cross-sectional survey design. Data were derived from the Research on the Employee Assistance Programme (EAP) for Young Talent in Tertiary Public Hospitals of Shandong Province, which aimed to develop feasible EAP interventions to enhance the motivation of healthcare workers and improve the quality of medical services. The survey was administered between August and November 2023. Participants were healthcare workers from provincial and municipal tertiary public hospitals.

Inclusion criteria were: (1) registered and currently employed physicians, nurses, and medical technicians; (2) at least one year of work experience; and (3) provision of informed consent and voluntary participation. Exclusion criteria included: (1) healthcare workers absent due to illness, maternity leave, or personal leave; and (2) visiting fellows, rotating physicians/nurses, and interns. According to Kendall’s sampling principle, the minimum sample size should be 5–10 times the number of variables included. With 77 items in the survey and using a 10-fold calculation, the minimum required sample was 770. The full English-language questionnaire used in this study, including all survey items and response options, is provided in Appendix 1. Allowing for 20% invalid or incomplete responses, the required sample size was at least 963.

For provincial hospitals, 11 institutions were selected by random sampling. For municipal hospitals, a multi-stage cluster random sampling strategy was adopted. Five prefecture-level cities were chosen according to socioeconomic development level: Qingdao and Jinan (high), Weifang and Linyi (medium), and Binzhou (low). One tertiary hospital was then randomly selected from each city (five hospitals in total), followed by probability proportional to size sampling. In total, 16 tertiary hospitals were included. Data collection was implemented following approval from the nursing administration departments of each hospital. Within each participating hospital, the questionnaire was distributed electronically to randomly selected eligible healthcare workers who met the inclusion criteria, rather than through an unrestricted open online survey. Local coordinators from the participating hospitals assisted with survey organization and dissemination. Participants completed the questionnaire anonymously on a digital platform after reading the study information and providing informed consent. The response rate was calculated based on the number of questionnaires distributed to eligible staff within the participating hospitals, rather than the total workforce of these hospitals. Of 1513 questionnaires distributed, 1503 valid responses were obtained (valid response rate: 99.3%), exceeding the minimum sample requirement. A total of 1503 healthcare workers were included in the final analysis. The sample was predominantly female (79.5%), nearly one-third were aged 31–35 years (31.1%), most were married (81.0%), and almost half had one child (45.0%). In terms of educational attainment, most participants held a bachelor’s degree (63.2%) or a master’s degree (22.0%). More detailed demographic and occupational characteristics are presented in Table 1. Informed consent was obtained from all subjects involved in the study.

**Table 1:** Characteristics of healthcare workers

Variables	Groups	N (%)	WE	*t*/*F*	*p* value
			Score (Mean ± SD)		
Total		1503 (100)	41.13 ± 17.02		
Gender	Male	308 (20.5)	41.81 ± 17.15	0.79	0.427
	Female	1195 (79.5)	40.95 ± 16.99		
Age (years)	≤25 years	155 (10.3)	41.77 ± 18.57	1.47	0.186
	26 ~ 30 years	144 (9.6)	40.42 ± 17.24		
	31 ~ 35 years	467 (31.1)	40.06 ± 17.18		
	36 ~ 40 years	422 (28.1)	41.31 ± 17.00		
	41 ~ 45 years	177 (11.8)	40.72 ± 15.94		
	46 ~ 50 years	67 (4.5)	44.30 ± 15.49		
	≥51 years	71 (4.7)	45.15 ± 15.65		
Marital status	Others^a^	286 (19.0)	40.94 ± 18.11	−0.21	0.833
	Married	1217 (81.0)	41.17 ± 16.76		
Parental status^b^	No children	85 (5.7)	41.92 ± 17.41	0.41	0.749
	One child	677 (45.0)	40.73 ± 16.67		
	Multiple children	472 (31.4)	41.73 ± 16.68		
	Unmarried without children	269 (17.9)	40.84 ± 18.38		
Education	Associate degree	66 (4.4)	40.09 ± 19.09	0.35	0.789
	Bachelor’s degree	950 (63.2)	40.89 ± 17.27		
	Master’s degree	330 (22.0)	41.63 ± 16.02		
	Doctoral degree	157 (10.4)	41.92 ± 16.69		
Hospital level	Grade A tertiary 3 hospital	1355 (90.2)	41.09 ± 17.12	−0.29	0.776
	Others^c^	148 (9.8)	41.51 ± 16.08		
Hospital type	General Hospital	1156 (76.9)	41.08 ± 17.01	−0.19	0.850
	Specialized Hospital	347 (23.1)	41.28 ± 17.08		
Occupation	Nurse	885 (58.9)	40.55 ± 16.67	1.38	0.251
	Doctor	410 (27.3)	41.72 ± 15.16		
	Others^d^	208 (13.8)	42.44 ± 21.37		
Years working	≤5 years	259 (17.2)	41.00 ± 17.58	1.14	0.335
	6–10 years	402 (26.7)	41.11 ± 16.83		
	11–15 years	441 (29.3)	40.13 ± 17.52		
	16–20 years	184 (12.2)	41.38 ± 16.66		
	≥21 years	217 (14.4)	43.13 ± 15.88		
Professional title	Junior title	484 (32.2)	41.48 ± 18.12	3.51	0.030
	Intermediate title	787 (52.4)	40.21 ± 16.80		
	Senior title	232 (15.4)	43.50 ± 15.10		
Employment type	Formal staff	655 (43.6)	41.32 ± 15.73	0.39	0.696
	Contract staff	848 (56.4)	40.98 ± 17.96		
Weekly working hours	<40 h	166 (11.0)	39.99 ± 18.19	0.72	0.538
	41-50 h	951 (63.3)	41.41 ± 17.06		
	51-60 h	253 (16.8)	41.60 ± 16.58		
	>60 h	133 (8.8)	39.66 ± 16.01		
Night shifts/month	0 day	504 (33.5)	41.34 ± 17.65	1.15	0.327
	1–2 days	210 (14.0)	39.42 ± 16.76		
	3–6 days	611 (40.7)	41.77 ± 16.98		
	≥7 days	178 (11.8)	40.33 ± 15.54		
Monthly income (CNY)	<5000	108 (7.2)	39.66 ± 18.52	1.49	0.203
	5000–10000	689 (45.8)	40.95 ± 17.23		
	10,001–15000	446 (29.7)	40.49 ± 16.56		
	15,001–20000	182 (12.1)	43.00 ± 16.27		
	>20000	78 (5.2)	44.01 ± 17.05		

**Note**

^a^ Includes those who have never been married, divorced, and widowed

^b^ Measured on the basis of marital status

^c^ Grade 3B and other Grade 3 hospitals

^d^ Includes medical technicians and pharmacists

**Abbreviations:** N, number; WE, work engagement; SD, standard deviation

### Measures

#### Perceived organizational support

POS was measured using the 24-item Chinese Employee Perceived Organizational Support Scale developed by Ling and colleagues [24], which was adapted from the original 36-item Survey of Perceived Organizational Support by Eisenberger and colleagues [14]. The scale includes three dimensions: work support, identifying value, and caring about well-being. All items were rated on a 5-point Likert scale ranging from 1 (“strongly disagree”) to 5 (“strongly agree”). The mean score of all items was calculated, with higher values indicating higher levels of POS. The scale has been widely used among Chinese employees and has shown good reliability and validity [25, 26]. In the present study, Cronbach’s α was 0.974.

#### Work engagement

Work engagement was assessed using the Utrecht Work Engagement Scale (UWES) developed by Schaufeli and colleagues [16] comprising three dimensions: vigor (6 items), dedication (4 items), and absorption (5 items). All 15 items were scored on a 7-point Likert scale. The Chinese version translated by Zhang and colleagues [27] has demonstrated good reliability and validity as well as its applicability in the Chinese population. Response options ranged from 0 (“never”) to 6 (“always”), yielding a total score of 0–90, with higher scores indicating greater work engagement. In this study, Cronbach’s α for the scale was 0.957.

#### Emotional labor

Emotional labor was assessed using the Emotional Labor Scale originally developed by Grandey [28] and subsequently translated and revised for use in Chinese healthcare settings by Luo and colleagues [29]. The scale comprises 14 items across three dimensions: surface acting, deep acting, and emotional display requirements. Surface acting reflects modifying outward emotional expressions without altering inner feelings; deep acting refers to actively regulating internal emotional states to align with organizational expectations; and emotional display requirements capture perceived organizational norms regarding emotional expression. All items were rated on a 6-point Likert scale ranging from 1 (strongly disagree) to 6 (strongly agree). Higher mean scores indicate higher levels of emotional labor. In this study, internal consistency was excellent, with a Cronbach’s α of 0.945.

#### Covariates

We collected a range of covariates that could influence work engagement among healthcare workers, covering sociodemographic and occupational characteristics. Sociodemographic variables included: sex (male/female), age (≤25, 26–30, 31–35, 36–40, 41–45, 46–50, ≥51 years), marital status (married/other [including single, divorced, widowed]), parenting status (none, one child, multiple children, unmarried without children), and education level (associate degree, bachelor’s, master’s, doctoral degree). Occupational variables included: hospital grade (Grade A class 3 hospital/other tertiary), hospital type (general/specialised), occupation (nurse/physician/other technical staff [including technicians and pharmacists]), years of work (≤5, 6–10, 11–15, 16–20, ≥21), professional title (junior/intermediate/senior), and employment type (permanent/contractual). Workload characteristics were also included as covariates: weekly working hours (<40h, 41–50h, 51–60h, >60h), monthly night shifts (0, 1–2, 3–6, ≥7), and monthly post-tax income (<¥5000, ¥5000–10 000, ¥10 001–15 000, ¥15 001–20 000, >¥20 000). All covariates were self-reported and treated as categorical variables in analyses to control for potential confounding.

### Statistical analysis

First, descriptive analyses were conducted to summarise sociodemographic and occupational characteristics and major study variables of healthcare workers. Continuous variables were presented as mean (SD), and categorical variables as frequency (percentage). Univariate analyses (*t* test, ANOVA, and correlation analysis) were then performed to explore associations between each variable and work engagement scores.

To identify latent POS subgroups among healthcare workers, LPA was performed based on the three POS dimensions: work support, identifying value, and caring about well-being. Model selection considered the Akaike information criterion (AIC), Bayesian information criterion (BIC), adjusted Bayesian information criterion (aBIC), entropy, the Vuong–Lo–Mendell–Rubin likelihood ratio test (VLMR), and the adjusted Lo–Mendell–Rubin likelihood ratio test (aLMR), as well as the bootstrap likelihood ratio test (BLRT), average posterior probabilities of assignment (APPA), together with class sizes and substantive interpretability. After the optimal solution was identified, participants were assigned to latent profiles according to their most likely class membership for subsequent analyses. Multiple ordinary least squares (OLS) regression models were then constructed in two steps to examine the associations of emotional labor and POS profiles with work engagement: first, unadjusted models; second, models adjusted for all covariates.

To test the moderating role of POS profiles in the emotional labor–work engagement relationship, interaction terms between emotional labor and POS profiles were included in the OLS models. Both unadjusted and adjusted interaction models were estimated, and simple slope analyses and graphical plotting were applied to interpret interaction effects.

In addition to the person-centered approach, we also examined POS as a continuous total score to test its moderating effect on the emotional labor–work engagement association. This variable-centered analysis was conducted for two reasons. First, it allowed direct comparison with prior studies that predominantly treated POS as a continuous construct. Second, it complemented the person-centered findings by assessing whether similar moderation patterns could also be observed when POS was operationalized using traditional composite scoring. Furthermore, to explore potential dimension-specific effects, separate interaction terms between emotional labor and each of the three POS dimensions (work support, identifying value, and caring about well-being) were also examined in OLS models. Restricted cubic spline (RCS) models were additionally fitted to examine potential nonlinear moderating effects of continuous POS on the association between emotional labor and work engagement.

Several sensitivity analyses were conducted to assess the robustness of the main findings. First, given the theoretical distinction among emotional labor dimensions, supplementary analyses were performed by replacing the overall emotional labor score with its three components—surface acting, deep acting, and emotional display requirements—in both the main-effect and interaction models, to examine whether the associations with work engagement and the moderating role of POS were consistent across dimensions. Second, because respondents were drawn from multiple hospital departments and information on hospital identifiers was not available due to the anonymous survey design, additional analyses were conducted using department as the clustering unit. Specifically, regression models were re-estimated with department fixed effects, and cluster-robust standard errors with CR2 adjustment were further applied at the department level. These sensitivity analyses were used to evaluate whether the main associations were robust to potential within-department non-independence and to alternative operationalisations of emotional labor.

All analyses were performed using R version 4.5.2 and Mplus version 8.8. Regression results are reported as coefficients, standard errors (SE), *t* values, *p* values, and 95% CIs. Model fit was assessed using R^2^ and adjusted R^2^. Statistical significance was set at *p* < 0.05.

### Ethics approval

The study was conducted in accordance with the Declaration of Helsinki and was approved by the Ethics Committee of Reproductive Hospital of Shandong University (Approval No. [2023]伦审字(116)号).

## Results

### Descriptive characteristics of the study sample

The demographic and occupational characteristics of the study sample are presented in Table 1. The mean work engagement score was 41.13 (SD 17.02), and the mean POS score was 77.35 (SD 19.74). Among the three POS dimensions, the mean scores were 32.11 (SD 9.20) for work support, 22.77 (SD 6.50) for identifying value, and 22.46 (SD 6.34) for caring about well-being. The corresponding item-level mean scores were 3.21 (SD 0.92), 3.25 (SD 0.93), and 3.21 (SD 0.91), respectively. Correlation analyses indicated that work engagement, emotional labor and all POS dimensions were significantly interrelated (all *p* < 0.001; Appendix 2 Table S1).

### Latent profile analysis of POS

Fit indices for the POS latent profile analysis are presented in Table 2. Lower values of AIC, BIC, and aBIC indicate better model fit, whereas higher entropy values (closer to 1.0) reflect clearer classification. Likelihood ratio tests (VLMR and aLMR) were statistically significant for the 2–5 class solutions (all *p* < 0.0001), suggesting that models with more classes fit better than those with one fewer class. In addition to these fit indices, Table 2 also presents class proportions, APPA, and class counts based on most likely class membership, which provide further information on classification quality and uncertainty. However, model selection was based not only on statistical fit but also on classification quality and substantive interpretability.

**Table 2:** Model fit indices and classification quality for latent profile analysis of POS

Classes	AIC	BIC	aBIC	Entropy	VLMR (P)	aLMR (P)	BLRT (P)	Class proportion	APPA	Class counts and proportions
1 class	12,038.468	12,070.359	12,051.299	–	–	–	–	1	1	1503 (1.000)
2 classes	10,599.716	10,652.868	10,621.101	0.754	<0.0001	<0.0001	<0.0001	0.469/0.531	0.923/0.930	704 (0.468)/799 (0.532)
3 classes	10,000.821	10,075.234	10,030.760	0.792	<0.0001	<0.0001	<0.0001	0.181/0.520/0.299	0.893/0.904/0.915	267 (0.178)/791 (0.526)/445 (0.296)
4 classes	9775.227	9870.901	9813.720	0.783	<0.0001	<0.0001	<0.0001	0.119/0.389/0.378/0.113	0.928/0.870/0.860/0.895	167 (0.111)/599 (0.399)/580 (0.386)/157 (0.104)
5 classes	9713.517	9830.452	9760.564	0.722	<0.0001	<0.0001	<0.0001	0.094/0.236/0.324/0.262/0.083	0.898/0.810/0.764/0.823/0.872	138 (0.092)/343 (0.228)/513 (0.341)/389 (0.259)/120 (0.080)

**Note**: Lower values of AIC, BIC, and aBIC indicate better relative model fit, whereas higher entropy values indicate clearer classification; entropy values of 0.80 or above are commonly considered to reflect good classification, and values close to or above 0.70 are generally considered acceptable. For VLMR, aLMR, and BLRT, a statistically significant *p* value suggests that the model with k classes provides a better fit than the model with k − 1 classes. APPA values closer to 1.0 indicate better classification precision, and values of 0.70 or higher are commonly considered acceptable. Class proportion refers to the model-estimated proportion of participants in each latent profile, whereas class counts and proportions refer to the observed sample distribution based on most likely latent class membership; these values may differ slightly because the latter are derived from individual modal assignment rather than estimated posterior class proportions. APPA refers to the average posterior probability of assignment for most likely class membership. For ease of interpretation, class proportion, APPA, and class counts are presented in ascending order of profile means within each model. BLRT results for some models should be interpreted with caution because Mplus indicated possible local maxima and a limited number of successful bootstrap draws

**Abbreviations:** AIC, Akaike information criterion; BIC, Bayesian information criterion; aBIC, adjusted Bayesian information criterion; VLMR (P), *p* value for the Vuong–Lo–Mendell–Rubin likelihood ratio test; aLMR (P), *p* value for the adjusted Lo–Mendell–Rubin likelihood ratio test; BLRT (P), *p* value for the bootstrap likelihood ratio test; APPA, average posterior probability of assignment

The 3-class solution was selected as the optimal model. First, compared with the 2-class model, the 3-class model showed a substantial improvement in information criteria (AIC/BIC/aBIC decreased) and a higher entropy (0.792 vs 0.754), indicating improved separation between profiles. Second, the 3-class model yielded well-balanced and practically meaningful class proportions (0.181/0.520/0.299), avoiding very small groups and supporting stable estimation and downstream regression analyses. For the selected 3-class solution, the APPA values were 0.893, 0.904, and 0.915, respectively, indicating acceptable classification quality. In contrast, although the 4- and 5-class models produced slightly lower AIC/BIC values, they did not improve classification quality (entropy decreased to 0.783 and 0.722, respectively) and introduced small classes (e.g., 11.3% and 11.9% in the 4-class model; 9.4% and 8.3% in the 5-class model), suggesting potential over-extraction and reduced practical interpretability. Therefore, the 3-class solution provided a parsimonious and interpretable representation of latent heterogeneity in POS while maintaining acceptable classification performance.

**Fig. 1:**
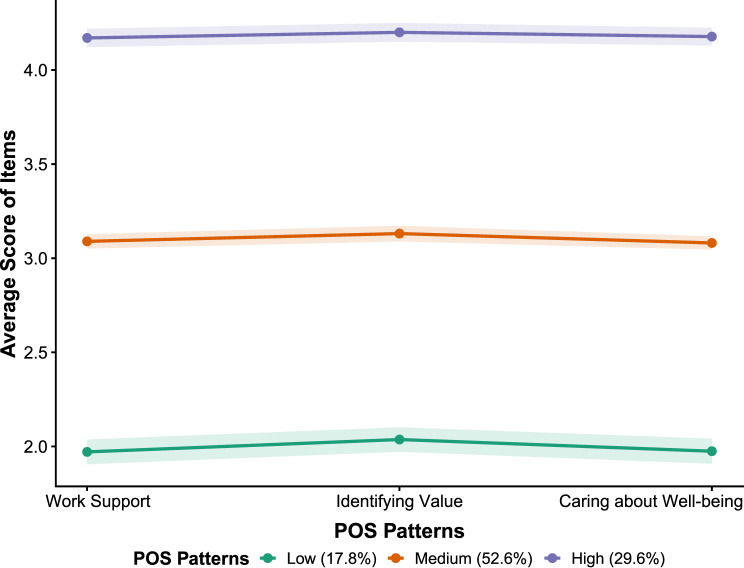
Latent profile of POS. Note: for all three dimensions of POS, a higher score indicates a higher level of POS. Abbreviations: POS, perceived organizational support

The characteristics of the three POS profiles are summarized in Appendix 2 Table S2. The distribution patterns of the three latent profiles across the POS dimensions are visually illustrated in Fig. 1. Across all three POS dimensions—work support, identifying value, and caring about well-being—mean item scores increased monotonically from the low POS profile (18.1%) to the medium POS profile (52.0%) and the high POS profile (29.9%). Post-hoc comparisons (Bonferroni-corrected) confirmed significant between-profile differences for each dimension (all *p* < 0.001), with a consistent ordering of high > medium >low. Accordingly, subsequent analyses were conducted using most likely class membership, and the profile-based associations were interpreted with awareness that some degree of classification uncertainty remained.

### Multivariate OLS regression of work engagement

Multivariate OLS regression results are presented in Table 3. The overall model explained a substantial proportion of variance in work engagement (R^2^ = 0.340; adjusted R^2^ = 0.323). Emotional labor was significantly negatively associated with work engagement (β = −0.35, *p* < 0.001), indicating that higher levels of emotional labor were linked to lower work engagement. Compared with healthcare workers in the low POS profile (reference group), those in the medium POS profile reported significantly higher work engagement (β = 10.65, *p* < 0.001), and those in the high POS profile demonstrated an even greater increase in work engagement (β = 21.74, *p* < 0.001).

**Table 3:** Multiple OLS regression analysis of WE

Variables	Adjusted model *β* (95% CI)	*p* value
Emotional labor	−0.35 (−0.41, −0.29)	<0.001
POS (ref: low)		
Medium	10.65 (8.61, 12.70)	<0.001
High	21.74 (19.34, 24.14)	<0.001
Gender (ref: male)		
Female	−0.29 (−2.37, 1.79)	0.787
Age (ref: ≤25 years)		
26–30 years	−2.41 (−6.27, 1.45)	0.221
31–35 years	−2.09 (−6.99, 2.82)	0.405
36–40 years	−1.51 (−7.05, 4.03)	0.594
41–45 years	−2.99 (−9.44, 3.46)	0.364
46–50 years	0.74 (−7.19, 8.66)	0.855
≥51 years	2.69 (−5.22, 10.59)	0.506
Marital status (ref: married)		
Others ^a^	0.56 (−6.26, 7.39)	0.871
Parental status ^b^ (ref: no children)		
One child	−3.10 (−6.54, 0.33)	0.077
Multiple children	−1.48 (−5.05, 2.09)	0.417
Unmarried	−3.20 (−10.80, 4.39)	0.409
Education (ref: associate degree)		
Bachelor’s degree	1.43 (−2.48, 5.35)	0.473
Master’s degree	2.58 (−1.95, 7.10)	0.265
Doctoral degree	0.22 (−5.15, 5.59)	0.936
Hospital level (ref: Grade A class 3 tertiary)		
Others^c^	−0.06 (−2.82, 2.71)	0.968
Hospital type (ref: general)		
Specialized hospital	−0.66 (−2.73, 1.40)	0.529
Position (ref: nurse)		
Doctor	−3.89 (−6.73, −1.06)	0.007
Others^d^	−3.67 (−6.19, −1.14)	0.004
Years working (ref: ≤5 years)		
6–10 years	1.59 (−2.06, 5.23)	0.394
11–15 years	1.17 (−3.12, 5.46)	0.594
16–20 years	2.15 (−3.00, 7.30)	0.414
≥21 years	1.15 (−5.16, 7.46)	0.722
Professional title (ref: junior)		
Intermediate	−2.32 (−4.56, −0.08)	0.043
Senior	−1.74 (−5.11, 1.63)	0.313
Employment type (ref: formal staff)		
Contract staff	1.37 (−0.85, 3.58)	0.228
Weekly working hours (ref: <40 h)		
41–50 h	2.01 (−0.35, 4.38)	0.096
51–60 h	1.55 (−1.35, 4.45)	0.294
>60 h	2.33 (−1.15, 5.81)	0.19
Night shifts/month (ref: 0 day)		
1–2 days	0.91 (−1.41, 3.24)	0.441
3–6 days	1.51 (−0.32, 3.33)	0.106
≥7 days	−0.25 (−2.84, 2.34)	0.852
Monthly income (ref: <5000)		
5000– 10,000	1.90 (−1.40, 5.19)	0.26
10,001– 15,000	1.66 (−1.87, 5.19)	0.358
15,001– 20,000	3.77 (−0.30, 7.84)	0.069
>20000	3.49 (−1.30, 8.29)	0.153
Constant	47.32 (36.94, 57.71)	<0.001
R^2^	0.34	
Adjusted R^2^	0.323	

**Note**

^a^ Includes those who have never been married, divorced, and widowed

^b^ Measured on the basis of marital status

^c^ Grade 3B and other Grade 3 hospitals

^d^ Includes medical technicians and pharmacists

**Abbreviations:** WE, work engagement; POS, perceived organizational support; CI, confidence interval; LB, lower bound; UB, upper bound

Among occupational characteristics, compared with nurses, doctors (β = −3.89, *p* = 0.007) and other technical staff (β = −3.67, *p* = 0.004) reported significantly lower work engagement. Additionally, healthcare workers with intermediate professional titles had lower work engagement compared with those holding junior titles (β = −2.32, *p* = 0.043). No significant associations were observed for sex, age, marital status, parental status, education level, hospital level, hospital type, years of work, employment type, working hours, night shifts, or income (all *p* > 0.05).

### Moderating effects of POS on the association between emotional labor and work engagement

**Fig. 2:**
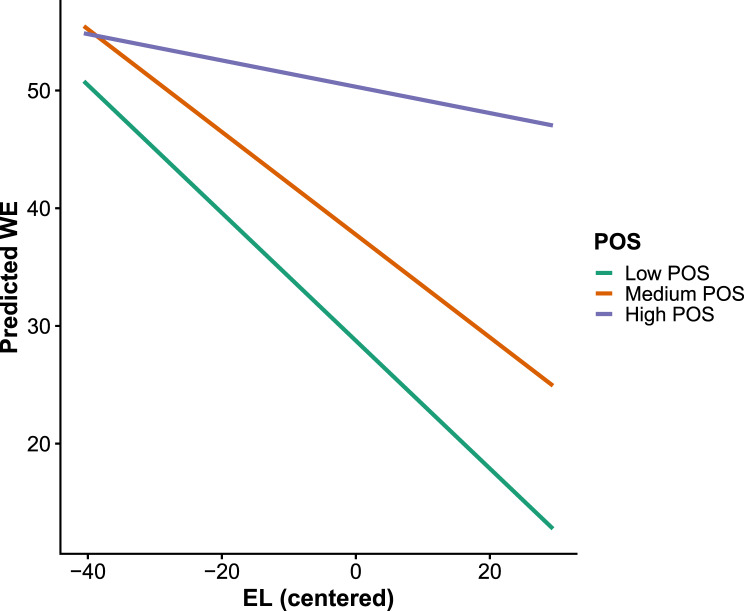
Moderating effect plots of different POS profiles. Abbreviations: POS, perceived organizational support; WE, work engagement; EL, emotional labor

Interaction effects between POS profiles and emotional labor are presented in Table 4. In the fully adjusted model, the interaction term for the high POS profile was significantly positive (β = 0.43, *p* < 0.001), indicating that high POS attenuated the negative association between emotional labor and work engagement. The interaction for the medium POS profile was not statistically significant (β = 0.11, *p* = 0.226). Figure 2 illustrates these moderating effects. Compared with the low POS profile, the negative slope between emotional labor and work engagement was substantially attenuated among individuals in the high POS profile, whereas the slope for the medium POS profile showed no significant buffering effect.

**Table 4:** Analysis of the interaction between the POS profiles and emotional labor

Variables	Unadjusted model *β* (95% CI)	*p* value	Adjusted model *β* (95% CI)	*p* value
Emotional labor^a^	−0.54 (−0.69, −0.39)	<0.001	−0.54 (−0.69, −0.39)	<0.001
POS (ref: Low)				
Medium	8.21 (5.84, 10.58)	<0.001	9.01 (6.63, 11.39)	<0.001
High	20.00 (17.35, 22.57)	<0.001	21.58 (18.91, 24.24)	<0.001
Interaction: EL × POS (ref: Low)				
EL × Medium	0.14 (−0.03, 0.31)	0.111	0.11 (−0.07, 0.28)	0.226
EL × High	0.42 (0.23, 0.60)	<0.001	0.43 (0.25, 0.62)	<0.001
Constant	31.63 (29.48, 33.79)	<0.001	28.74 (19.26, 38.22)	<0.001
R^2^	0.324		0.353	
Adjusted R^2^	0.322		0.335	

**Note**: Adjusted model controlled for gender, age, marital status, parental status, education, hospital level, hospital type, position, years working, professional title, employment type, weekly working hours, night shifts/month, and monthly income

^a^ Coefficients are rounded to two decimal places; small differences between the unadjusted and adjusted models may not be visible due to rounding

**Abbreviations:** POS, perceived organizational support; CI, confidence interval

Supplementary analyses yielded consistent findings. Significant positive interactions were observed for work support (β = 0.105, *p* < 0.001), identifying value (β = 0.142, *p* < 0.001), and caring about well-being (β = 0.133, *p* < 0.001) (Appendix 2 Table S3). Similarly, when POS was operationalized as a continuous total score, the interaction between emotional labor and POS remained significant (β = 0.0063, *p* < 0.001; Appendix 2 Table S4), supporting the robustness of the moderating effect.

**Fig. 3:**
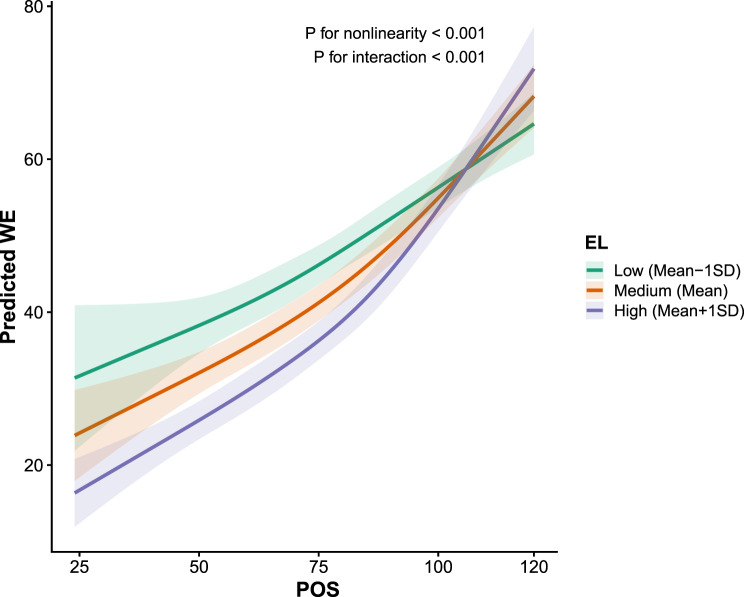
Restricted cubic spline analysis of the moderating effect of POS on the association between EL and WE. Note: the solid line represents the estimated association between emotional labor and work engagement across levels of POS, and the shaded area indicates the 95% confidence interval. Restricted cubic spline models were adjusted for covariates. Abbreviations: EL, emotional labor; WE, work engagement; POS, perceived organizational support, SD, standard deviation

RCS analysis further examined the moderating effect of continuous POS on the association between emotional labor and work engagement (Fig. 3). After adjustment for covariates, both the nonlinearity test and the interaction test were statistically significant (both *p* < 0.001), indicating potential nonlinearity in the moderating effect of POS. As shown in Fig. 3, the negative association between emotional labor and work engagement was generally attenuated at higher levels of POS, with the buffering effect becoming more evident as POS increased. Together, these findings consistently support a buffering role of POS in mitigating the detrimental effect of emotional labor on work engagement.

### Sensitivity analyses

Several sensitivity analyses were conducted to assess the robustness of the main findings. When emotional labor was analysed separately as surface acting, emotional display requirements, and deep acting, all three dimensions were significantly negatively associated with work engagement in the adjusted main-effect models (all *p* < 0.001; Appendix 2 Tables S5–S7). In the interaction models, the interaction terms between each dimension and the high POS profile were consistently positive and statistically significant, whereas those for the medium POS profile were not significant.

Additional department-level sensitivity analyses yielded similar results. After further adjustment for department fixed effects, emotional labor remained negatively associated with work engagement, and the interaction between emotional labor and the high POS profile remained significant, whereas the interaction for the medium POS profile remained non-significant (Appendix 2 Table S8). Similar findings were observed when department-clustered CR2 robust standard errors were applied (Appendix 2 Table S9). Overall, these results supported the robustness of the main findings.

## Discussion

Using data from 1503 healthcare workers in China, this study identified three distinct profiles of POS through latent profile analysis: low POS (18.1%), medium POS (52.0%), and high POS (29.9%). Emotional labor was significantly negatively associated with work engagement. Notably, the negative association between emotional labor and work engagement was substantially attenuated among the high-POS group, whereas no significant buffering effect was observed in the medium-POS group. These findings suggest that healthcare workers’ perceptions of organizational support exhibit a stratified structure. Enhancing organizational support, particularly for employees experiencing low levels of support, may help sustain work engagement in high-demand healthcare environments.

The three latent POS profiles identified in this study—low POS (18.1%), medium POS (52.0%), and high POS (29.9%)—indicate clear group stratification in healthcare workers’ perceptions of organizational support. These findings enrich the empirical foundation of Organizational Support Theory (OST) from a population heterogeneity perspective. A meta-analysis has also confirmed that POS demonstrates stable and widespread predictive effects, with its formation influenced by multiple factors, including leadership behaviors, human resource practices, and working conditions [30]. However, most previous studies have adopted variable-centered approaches that treat POS as a continuous variable and have paid limited attention to potential structural subgroups within employees’ perceptions of support. In recent years, scholars have increasingly called for person-centered approaches to reveal the underlying heterogeneity in POS [31]. For example, a growth mixture modeling study has shown that employees can be categorized into distinct trajectories of POS, such as stable-high, stable-low, and changing patterns, suggesting that perceptions of support exhibit stratified characteristics over time [31]. Similarly, the present study identified three POS profiles among healthcare workers in tertiary hospitals in China, suggesting that POS also presents a stratified structure in cross-sectional contexts.

Previous studies have also applied latent class approaches to reveal stratified distributions of organizational support or organizational resources among employees across various contexts, including public health institutions and corporate organizations [32, 33]. In this context, the proportional structure identified in the present study carries important theoretical implications. Nearly one-third of healthcare workers developed a stable and positive perception of organizational support, which is consistent with the “cumulative social exchange mechanism” emphasized by Organizational Support Theory (OST), whereby sustained positive treatment from the organization strengthens employees’ overall evaluation of organizational support [15]. Meanwhile, the low-POS group still accounted for 18.1%, a proportion that should not be overlooked, suggesting that a segment of healthcare workers remains in a long-term state of insufficient support under high-pressure medical environments and may therefore face elevated risks related to work motivation and mental health. Overall, the present study confirms the structural heterogeneity of POS in the Chinese healthcare context and provides a stratified perspective for understanding the differentiated effects of POS on outcome variables such as work engagement.

This study further supports the applicability of Organizational Support Theory [14] and the JD–R model [5–8] among healthcare workers. Specifically, emotional labor, originally conceptualized as the regulation of feelings and emotional expressions in accordance with occupational display rules [1, 2], may be understood as an important job demand in healthcare work and was negatively associated with work engagement in the present study, whereas organizational support, as a key job resource, was positively associated with work engagement and appeared to attenuate the negative association between emotional labor and work engagement to some extent. This finding is consistent with the central assumptions of the JD–R model, which posits that job resources can enhance engagement through motivational pathways while also mitigating the depletion effects caused by high job demands [8]. From a theoretical perspective, this buffering mechanism can be explained through social exchange theory and conservation of resources theory [34, 35]. When healthcare workers perceive that the organization invests in emotional support, resource provision, and career development opportunities, they may reciprocate through stronger commitment and engagement. At the same time, such support can replenish psychological resources, thereby alleviating the exhaustion associated with emotional labor.

Furthermore, this study reveals a boundary effect of POS in the relationship between emotional labor and work engagement. Specifically, the negative effect of emotional labor on work engagement was significantly attenuated in the high-POS group, whereas employees in the low-POS group were more vulnerable to its adverse impact. This finding suggests that organizational support not only functions as a direct promotive factor but also serves as an important protective resource in high-demand work contexts. From the perspective of the JD–R model, individuals with abundant resources are more likely to adopt adaptive self-regulation strategies to cope with the psychological demands associated with emotional labor, whereas those with limited external resources may be more likely to fall into cycles of resource depletion. Conservation of resources theory further posits that individuals with fewer resources are more susceptible to additional stressors, while those with greater resource reserves possess stronger recovery capacities [35]. Therefore, healthcare workers in the high-POS group may be better able to cope with emotional labor demands without experiencing substantial declines in work engagement.

In addition, the continuous interaction analysis and restricted cubic spline models suggest that the buffering effect of POS may exhibit potential nonlinearity, indicating that the protective effect of support may stabilize once support levels reach a certain threshold. This finding provides more refined insights into the underlying mechanisms and is consistent with previous research highlighting the heterogeneity and complex structure of POS [31]. From a motivational perspective, POS enhances employees’ sense of belonging and identifying value, thereby strengthening perceived work meaning and organizational identification and promoting the maintenance of intrinsic motivation [15]. When support levels are low, individuals may struggle to develop stable psychological safety and value identification, making emotional labor more likely to be experienced as a burden. In contrast, when support levels are high, stronger organizational trust and emotional connections may facilitate more positive coping with emotional regulation demands.

The present study has important implications for hospital management and health policy. In healthcare settings characterized by high emotional labor demands, organizational support not only promotes work engagement but also buffers psychological strain among healthcare workers. Strengthening organizational support should therefore be considered a key component of workforce management and occupational health strategies in hospitals. Potential approaches include improving performance feedback systems, enhancing communication transparency, providing psychological support services, and strengthening career development opportunities. Evidence from participatory organizational interventions in emergency departments suggests that involving employees in the co-design of work processes and environments can reduce perceived stress and improve psychosocial working conditions [36]. From a resource allocation perspective, hospitals may benefit from prioritizing employees with lower levels of POS. Targeted strategies such as regular managerial communication, emotional support training, and team-building initiatives may help prevent emotional labor from translating into sustained motivational decline among this group, while efforts for employees with medium support levels should focus on strengthening the stability and visibility of existing support mechanisms to facilitate progression toward higher levels of perceived support. Emotional labor and insufficient organizational support have increasingly been recognized as common challenges affecting healthcare workforce engagement and well-being across healthcare systems worldwide [37]. Given the growing pressures on healthcare workers, systematically strengthening organizational support may represent an important strategy for sustaining workforce engagement and improving healthcare service quality. The stratified support structure identified in this study provides useful insights for designing targeted organizational interventions aimed at promoting workforce well-being and health system performance.

This study has several limitations. First, the cross-sectional design limits the ability to establish causal relationships. Although theoretical models suggest that emotional labor may influence work engagement through resource-related mechanisms, future longitudinal or intervention studies are needed to further verify the buffering effect of organizational support. Second, the sample was drawn from tertiary hospitals in Shandong Province, and the external validity of the findings should be further examined in different regions, healthcare institution levels, and international healthcare contexts. Third, the study relied primarily on self-reported questionnaires, which may introduce common method bias. Moreover, although the survey was anonymous and voluntary, it was administered within participating hospitals, and this organizationally facilitated procedure may have contributed to the high valid response rate. Accordingly, the possibility of response pressure or participation bias cannot be entirely ruled out. In addition, because hospital identifiers were not collected in the anonymous survey, we were unable to directly assess between-hospital variance, calculate hospital-level intraclass correlation coefficients, or fit multilevel models at the hospital level. Although supplementary analyses using department fixed effects and department-clustered CR2 robust standard errors yielded similar results, residual clustering at the hospital level cannot be completely excluded. Future studies may incorporate supervisor ratings, peer evaluations, objective performance indicators, more independent data collection procedures, and explicit organizational identifiers to strengthen the robustness of the findings. Fourth, although the selected 3-class solution showed acceptable classification quality, with an entropy of 0.792 and APPA values above 0.89, some degree of classification uncertainty remained. In the present study, subsequent regression and moderation analyses were conducted using most likely class assignment, which is common in applied latent profile research but does not fully account for classification error. As a result, the observed between-profile differences and moderating effects may have been attenuated to some extent, and the estimated associations should therefore be interpreted with appropriate caution. Future studies could further strengthen inference by applying three-step approaches or other methods that explicitly account for classification uncertainty when examining distal associations of latent profiles. Finally, this study mainly focused on organizational support as a moderating factor and did not systematically examine other potential organizational resources, such as team support, leadership support, or organizational climate. For example, previous longitudinal research has shown that psychosocial safety climate (PSC) may vary across occupational groups and change over time among healthcare workers [38], providing a useful reference for incorporating organizational climate variables into extended models. Future research could further develop multilevel models to examine how organizational resources at different levels jointly buffer the effects of emotional labor, thereby providing a more comprehensive understanding of the mechanisms shaping work motivation and occupational health in healthcare settings.

## Conclusion

In this cross-sectional study of healthcare workers in Chinese tertiary hospitals, POS exhibited clear latent heterogeneity and was associated with a buffering pattern in the association between emotional labor and work engagement. These findings highlight organizational support as an important and potentially modifiable resource for sustaining healthcare workforce engagement in high-demand clinical environments. Strengthening organizational support systems, particularly for employees with low levels of perceived support, may help alleviate the adverse association of emotional labor with work engagement among healthcare workers. Future research using longitudinal and intervention designs is needed to further clarify causal pathways and examine how organizational support strategies can be effectively implemented to enhance workforce resilience in healthcare settings.

## Electronic supplementary material

Below is the link to the electronic supplementary material.

Supplementary Material 1

Supplementary Material 2

## Data Availability

The datasets analyzed during the current study are not publicly available but are available from the corresponding author on reasonable request.

## References

[1] Hochschild AR. Emotion work, feeling rules, and social structure. Am J Sociol. 1979;85(3):551–75. 10.1086/227049.

[2] Hochschild AR. The managed heart: commercialization of human feeling. Berkeley: University of California Press; 2012.

[3] Larson EB. Clinical empathy as emotional labor in the patient-physician relationship. JAMA. 2005;293(9):1100–06. 10.1001/jama.293.9.1100. 15741532 10.1001/jama.293.9.1100

[4] Vinson AH, Underman K. Clinical empathy as emotional labor in medical work 1. In: Sociology through emotions. edn: Routledge; 2025. p. 165–86.

[5] Demerouti E, Bakker AB, Nachreiner F, Schaufeli WB. The job demands-resources model of burnout. J Educ Chang Appl Phychol. 2001;86(3):499. 10.1037/0021-9010.86.3.499. 11419809

[6] Bakker AB, Demerouti E. The job demands-resources model: state of the art. J Managerial Phychol. 2007;22(3):309–28. 10.1108/02683940710733115.

[7] Xanthopoulou D, Bakker AB, Demerouti E, Schaufeli WB. Reciprocal relationships between job resources, personal resources, and work engagement. J Vocational Behav. 2009;74(3):235–44. 10.1016/j.jvb.2008.11.003.

[8] Bakker AB, Demerouti E. Job demands–resources theory: taking stock and looking forward. J Occup Health Phychol. 2017;22(3):273. 10.1037/ocp0000056. 10.1037/ocp0000056 27732008

[9] Chen CC, Lan YL, Chiou SL, Lin YC. The effect of emotional labor on the physical and mental Health of Health professionals: emotional exhaustion has a mediating effect. Healthcare (Basel, Switz). 2022;11(1):104. 10.3390/healthcare11010104. 10.3390/healthcare11010104 PMC9819436 36611564

[10] Psilopanagioti A, Anagnostopoulos F, Mourtou E, Niakas D. Emotional intelligence, emotional labor, and job satisfaction among physicians in Greece. BMC Health Serv Res. 2012;12(1):463. 10.1186/1472-6963-12-463. 23244390 10.1186/1472-6963-12-463 PMC3541956

[11] Jeung DY, Kim C, Chang SJ. Emotional labor and burnout: a review of the literature. Yonsei Med J. 2018;59(2):187–93. 10.3349/ymj.2018.59.2.187. 29436185 10.3349/ymj.2018.59.2.187 PMC5823819

[12] West CP, Tan AD, Habermann TM, Sloan JA, Shanafelt TD. Association of resident fatigue and distress with perceived medical errors. JAMA. 2009;302(12):1294–300. 19773564 10.1001/jama.2009.1389

[13] Jolly PM, Kong DT, Kim KY. Social support at work: an integrative review. J Organ Behav. 2021;42(2):229–51. 10.1002/job.2485.

[14] Eisenberger R, Huntington R, Hutchison S, Sowa D. Perceived organizational support. J Educ Chang Appl Phychol. 1986;71(3):500. 10.1037/0021-9010.71.3.500.

[15] Eisenberger R, Rhoades Shanock L, Wen X. Perceived organizational support: why caring about employees counts. Annu Rev Organ Psychol Organ Behav. 2020;7(1):101–24. 10.1146/annurev-orgpsych-012119-044917.

[16] Schaufeli WB, Bakker AB. Utrecht work engagement scale: preliminary manual. Occup Health (Lond) Phychol Unit, Utrecht Univ, Utrecht. 2003;26(1):64–100.

[17] Rockstuhl T, Eisenberger R, Shore LM, Kurtessis JN, Ford MT, Buffardi LC, et al. Perceived organizational support (POS) across 54 nations: a cross-cultural meta-analysis of POS effects. J Int Bus Stud. 2020;51(6):933–62. 10.1057/s41267-020-00311-3.

[18] Li M, Chen R, Liu Q, Yang M, Li B, Li H, et al. The relationship between perceived organizational support and presenteeism among pediatric nurses in China: professional identity plays a mediating role. BMC Nurs. 2026;25(1). 10.1186/s12912-026-04347-y. Epub ahead of print. 10.1186/s12912-026-04347-y PMC13049763 41761213

[19] Pattali S, Sankar JP, Al Qahtani H, Menon N, Faizal S. Effect of leadership styles on turnover intention among staff nurses in private hospitals: the moderating effect of perceived organizational support. BMC Health Serv Res. 2024;24(1):199. 10.1186/s12913-024-10674-0. 38355546 10.1186/s12913-024-10674-0 PMC10865721

[20] Zheng J, Feng S, Gao R, Gong X, Ji X, Li Y, et al. The relationship between organizational support, professional quality of life, decent work, and professional well-being among nurses: a cross-sectional study. BMC Nurs. 2024;23(1):425. 10.1186/s12912-024-02114-5. 38918776 10.1186/s12912-024-02114-5 PMC11197337

[21] Morin AJS, Morizot J, Boudrias J-S, Madore I. A multifoci person-centered perspective on workplace affective commitment: a latent profile/factor mixture analysis. Organ Res Methods. 2011;14(1):58–90. 10.1177/1094428109356476.

[22] Wang M, Hanges PJ. Latent class procedures: applications to organizational research. Organ Res Methods. 2011;14(1):24–31. 10.1177/1094428110383988.

[23] Caesens G, Morin AJS, Gillet N, Stinglhamber F. Perceived support profiles in the workplace: a longitudinal perspective. Group Organ Manage. 2023;48(3):833–73. 10.1177/10596011211044581.

[24] Ling W, Yang H, Fang L. Perceived organizational support of enterprise employees. Acta Psychologica Sin. 2006;38(2):281–87 (in Chinese.

[25] Tian L, Wu A, Li W, Huang X, Ren N, Feng X, et al. Relationships between perceived organizational support, psychological capital and work engagement among Chinese infection control nurses. RMHP. 2023;Volume 16:551–62. 10.2147/RMHP.S395918. 10.2147/RMHP.S395918 PMC10081527 37035271

[26] Zhu C, Xia M, Xie H, Wang Y, Ye J, Xu J. Primary school teachers’ perceived organizational support and job satisfaction: the mediating role of collective efficacy. Soc Behav Pers. 2024;52(2):1–9. 10.2224/sbp.12915.

[27] Zhang Y, Gan Y. Reliability and validity of the Chinese version of the Utrecht work engagement scale (UWES). Chin J Clin Phychol. 2005;13(3):268–70, 281 (in Chinese.

[28] Grandey AA. When “the show must go on”: surface acting and deep acting as determinants of emotional exhaustion and peer-rated service delivery. Acad Manage J. 2003;46(1):86–96. 10.2307/30040678.

[29] Luo H, Sun Q, Gu L. Effects of nurses’ emotional labor ability on job burnout. Chin J Nurs. 2008;43(11):969–71 (in Chinese.

[30] Kurtessis JN, Eisenberger R, Ford MT, Buffardi LC, Stewart KA, Adis CS. Perceived organizational support: a meta-analytic evaluation of organizational support theory. J Manage. 2017;43(6):1854–84. 10.1177/0149206315575554.

[31] Caesens G, Morin AJS, Stinglhamber F. Longitudinal trajectories of perceived organizational support: a growth mixture analysis. JMP. 2020;35(6):481–95. 10.1108/JMP-01-2020-0027.

[32] Hu H, Allen P, Yan Y, Reis RS, Jacob RR, Brownson RC. Organizational supports for research evidence use in state public health agencies: a latent class analysis. J Public Health Manag Pract. 2019;25(4):373–81. 10.1097/PHH.0000000000000821. 31136511 10.1097/PHH.0000000000000821 PMC6269222

[33] Ergun E, Tunca S, Cetinkaya G, Balcıoğlu YS. Exploring the roles of work engagement, psychological empowerment, and perceived organizational support in innovative work behavior: a latent class analysis for sustainable organizational practices. Sustainability. 2025;17(4):1663. 10.3390/su17041663.

[34] Cropanzano R, Mitchell MS. Social exchange theory: an interdisciplinary review. J Manage. 2005;31(6):874–900. 10.1177/0149206305279602.

[35] Hobfoll SE, Halbesleben J, Neveu J-P, Westman M. Conservation of resources in the organizational context: the reality of resources and their consequences. Annu Rev Organ Psychol Organ Behav. 2018;5(1):103–28. 10.1146/annurev-orgpsych-032117-104640.

[36] Van Simaeys H, Mannaerts L, Serraes B, Clays E. Clays E: evaluating the impact of a participatory organizational intervention on reducing occupational stress in an emergency department setting: a one group pretest-posttest design. BMC Public Health. 2025;25(1):2480. 10.1186/s12889-025-23540-3. 40676572 10.1186/s12889-025-23540-3 PMC12269240

[37] Van Den Berg JW, Mastenbroek NJJM, Scheepers RA, Jaarsma ADC. Work engagement in health professions education. Med Teach. 2017;39(11):1110–18. 10.1080/0142159X.2017.1359522. 28830279 10.1080/0142159X.2017.1359522

[38] Abu Bakar N, Bulgiba A, Isahak M. Perceived psychosocial safety climate (PSC) level and its association with occupational outcomes among clinical unit healthcare workers in a Malaysian hospital: a three-wave longitudinal study. BMC Public Health. 2025;25(1):1418. 10.1186/s12889-025-22562-1. 40234778 10.1186/s12889-025-22562-1 PMC12001723

