# Supplementary material for: Perceived organizational support profiles and their moderating role in the association between emotional labor and work engagement among Chinese healthcare workers: a latent profile analysis
Source: BMC Health Serv Res. 2026 May 9;26:904. doi: 10.1186/s12913-026-14626-8 (PMC13326138; doi:10.1186/s12913-026-14626-8)
Supplement: Supplementary file 1 — Supplementary Material 1 [file 12913_2026_14626_MOESM1_ESM.docx]

# Survey on Employee Assistance Programs for Young Healthcare Professionals in Tertiary Public Hospitals in Shandong Province: An Emotional Labor Perspective

**Dear Sir/Madam,**

Hello! Thank you for participating in this survey. The purpose of this study is to understand healthcare workers’ **levels of emotional labor, work engagement, and perceived organizational support**, and to explore the relationships among these factors. The findings are expected to provide evidence for improving the working environment of healthcare professionals and promoting their physical and mental well-being.

This questionnaire is **anonymous**, and all information will be used solely for academic research. The results will be reported in an aggregated statistical form, and no personal information will be disclosed. Therefore, please feel free to answer honestly. Completing the questionnaire will take approximately **5–10 minutes**. There are no right or wrong answers; please select the option that best reflects **your actual experience and first impression**.

Your valuable responses are very important to this research. We sincerely appreciate your support and cooperation.

**We wish you success in your work!**

**Part I. Demographic and Occupational Characteristics**

**1. What is your gender?** [Single choice]

○ Male
○ Female

**2. Please indicate your age group.** [Single choice]

○ 25 years or younger
○ 26–30 years
○ 31–35 years
○ 36–40 years
○ 41–45 years
○ 46–50 years
○ 51 years or older

**3. What is your current marital status?** [Single choice]

○ Single
○ Married
○ Divorced
○ Widowed
○ Other: __________

**4. How many children do you currently have?** [Single choice]

○ None
○ One
○ Two
○ Three or more

This question applies only to respondents who selected “Married,” “Divorced,” “Widowed,” or “Other” in Question 3.

**5. What is the highest level of education you have completed?** [Single choice]

○ Technical secondary school
○ Junior college (associate degree)
○ Bachelor’s degree
○ Master’s degree
○ Doctoral degree

**6. What is the administrative level of the hospital where you currently work?** [Single choice]

○ Secondary Grade B or below
○ Secondary Grade A
○ Tertiary Grade B
○ Tertiary Grade A
○ Other: __________

**7. What type of hospital do you currently work in?** [Single choice]

○ General hospital
○ Specialized hospital

**8. Which department do you currently work in?** [Single choice]

○ Internal Medicine
○ Surgery
○ Obstetrics and Gynecology
○ Pediatrics
○ Otorhinolaryngology (ENT)
○ Traditional Chinese Medicine
○ Emergency Department
○ Intensive Care Unit (ICU)
○ Operating Room
○ Outpatient Department
○ Medical Technology Department
○ Other: __________

**9. What is your current professional role in the hospital?** [Single choice]

○ Physician
○ Nurse
○ Medical technician
○ Pharmacist
○ Other: __________

**10. How many years have you been working in the healthcare profession?** [Single choice]

○ 5 years or less
○ 6–10 years
○ 11–15 years
○ 16–20 years
○ 21–25 years
○ 26–30 years
○ 31 years or more

**11. What is your current professional title?** [Single choice]

○ Junior title
○ Intermediate title
○ Associate senior title
○ Senior title

**12. Do you currently hold any administrative or managerial position?** [Multiple choice]

○ None
○ Teaching group leader
○ Clinical group leader
○ Deputy head nurse
○ Head nurse
○ Chief nurse
○ Director / Deputy Director of Nursing Department
○ Other: __________

This question applies only to respondents who selected “Nurse” in Question 9.

**13. Do you currently hold any administrative or managerial position?** [Multiple choice]

○ None
○ Teaching group leader
○ Clinical group leader
○ Deputy department director
○ Department director
○ Vice president / President of the hospital
○ Other: __________

This question applies only to respondents who selected “Physician,” “Medical technician,” “Pharmacist,” or “Other” in Question 9.

**14. What is your current employment status?** [Single choice]

○ Permanent position (official establishment)
○ Personnel agency employment
○ Contract-based employment
○ Other: __________

**15. On average, how many hours do you work per week in the hospital?** [Single choice]

○ Less than 40 hours
○ 40–50 hours
○ 50–60 hours
○ More than 60 hours

**16. How many night shifts do you usually work per month?** [Single choice]

○ 0
○ 1–2
○ 3–4
○ 5–6
○ 7–8
○ 9 or more

**17. What is your approximate monthly income (RMB)?** [Single choice]

○ Less than 5,000
○ 5,000–10,000
○ 10,001–15,000
○ 15,001–20,000
○ More than 20,000

**Part II. Emotional Labor Scale**

**Instruction:**
Please read the following statements carefully. Indicate the extent to which you agree with each statement.

1 = Strongly disagree
2 = Disagree
3 = Slightly disagree
4 = Slightly agree
5 = Agree
6 = Strongly agree

| **Item** |
| --- |
| 1. Displaying appropriate expressions and attitudes at work feels like acting to me. |
| 2. The emotions I show to patients are different from what I actually feel inside. |
| 3. In order to serve patients appropriately, I have learned to pretend certain emotions. |
| 4. When interacting with patients, I display the emotions required by my job without changing my inner feelings. |
| 5. In order to present specific expressions and attitudes to patients, I hide my true feelings. |
| 6. I merely pretend to have the emotions that are required in my job. |
| 7. My job requires me to display emotions that differ from my genuine feelings. |
| 8. The hospital considers my work attitude when evaluating performance. |
| 9. The hospital requires me to display specific behaviors or facial expressions to convey the image it wants to present. |
| 10. When dealing with different patients, the hospital requires me to display different emotional expressions. |
| 11. Even when patients make unreasonable requests, the hospital requires me to remain friendly and polite. |
| 12. At work, I try to overcome my negative emotions and sincerely provide patients with a friendly attitude. |
| 13. When I feel unhappy, I temporarily put aside unpleasant feelings so that I can maintain a positive mood when interacting with patients. |
| 14. I try to genuinely feel the emotions that I should express to patients. |

**Part III. Work Engagement Scale (UWES-17)**

**Instruction:**
The following 17 statements are about how you feel at work. Please read each statement carefully and decide if you ever feel this way about your job.

0 = Never
1 = Almost never (a few times a year)
2 = Rarely (once a month or less)
3 = Sometimes (a few times a month)
4 = Often (once a week)
5 = Very often (a few times a week)
6 = Always (every day)

| **Item** |
| --- |
| 1. At my work, I feel bursting with energy |
| 2. I find the work that I do full of meaning and purpose |
| 3. Time flies when I'm working |
| 4. At my job, I feel strong and vigorous |
| 5. I am enthusiastic about my job |
| 6. When I am working, I forget everything else around me |
| 7. My job inspires me |
| 8. When I get up in the morning, I feel like going to work |
| 9. I feel happy when I am working intensely |
| 10. I am proud on the work that I do |
| 11. I am immersed in my work |
| 12. I can continue working for very long periods at a time |
| 13. To me, my job is challenging |
| 14. I get carried away when I'm working |
| 15. At my job, I am very resilient, mentally |
| 16. It is difficult to detach myself from my job |
| 17. At my work I always persevere, even when things do not go well |

**Part IV. Perceived Organizational Support Scale**

**Instruction:**
The following statements describe your perception of organizational support in your workplace. Please indicate the extent to which you agree with each statement.

1 = Strongly disagree
2 = Disagree
3 = Uncertain
4 = Agree
5 = Strongly agree

| **Item** |
| --- |
| 1. My organization recognizes employees who perform well. |
| 2. My organization does not take advantage of employees whenever possible. |
| 3. My organization agrees to reasonable requests to change working conditions. |
| 4. My organization values employees’ work goals and values. |
| 5. My organization provides help when I encounter problems at work. |
| 6. My organization assigns employees to the most suitable work. |
| 7. My organization provides opportunities for promotion. |
| 8. My organization makes work interesting for employees. |
| 9. My organization helps employees realize their work potential. |
| 10. My organization values employees’ opinions at work. |
| 11. My organization believes that retaining employees benefits the organization. |
| 12. My organization attempts to retain employees who intend to leave. |
| 13. My organization considers dismissing employees a significant loss. |
| 14. My organization does not easily dismiss employees. |
| 15. My organization prefers reassignment rather than dismissal. |
| 16. My organization takes pride in employees’ achievements. |
| 17. My organization allows laid-off employees to return when possible. |
| 18. My organization rewards employees for extra effort. |
| 19. My organization understands employees’ occasional absence for personal reasons. |
| 20. My organization provides special help to employees when needed. |
| 21. My organization cares about employees’ life conditions. |
| 22. My organization increases employees’ salaries when profits are high. |
| 23. My organization actively considers appropriate salary levels for employees. |
| 24. My organization considers employees’ interests when making decisions. |
