# Supplementary material for: Perceived organizational support profiles and their moderating role in the association between emotional labor and work engagement among Chinese healthcare workers: a latent profile analysis
Source: BMC Health Serv Res. 2026 May 9;26:904. doi: 10.1186/s12913-026-14626-8 (PMC13326138; doi:10.1186/s12913-026-14626-8)
Supplement: Supplementary file 2 — Supplementary Material 2 [file 12913_2026_14626_MOESM2_ESM.docx]

**Supplementary Appendix 2**

Supplement to: **Profiles of perceived organizational support and their moderating role between emotional labor and work engagement among Chinese healthcare workers**

**Contents**

[Table S1 Correlation analysis 2](#_Toc13076)

[Table S2 Comparison of the average scores of each dimension item of different profiles of POS 2](#_Toc19975)

[Table S3 Analysis of the interaction between the POS dimensions and emotional labor 2](#_Toc14443)

[Table S4 Analysis of the interaction between the total POS score and emotional labor 3](#_Toc19633)

[Table S5. Main and interaction effects of surface acting on work engagement across perceived organizational support profiles 3](#_Toc17917)

[Table S6. Main and interaction effects of emotional display requirements on work engagement across perceived organizational support profiles 4](#_Toc26228)

[Table S7. Main and interaction effects of deep acting on work engagement across perceived organizational support profiles 4](#_Toc5099)

[Table S8. Sensitivity analyses of the association between emotional labor and work engagement after additional adjustment for department fixed effects 5](#_Toc802)

[Table S9. Sensitivity analyses of the association between emotional labor and work engagement using department-clustered CR2 robust standard errors 5](#_Toc8723)

# Table S1 Correlation analysis

|  | WE | Emotional labor | Work support | Identifying value | Caring about well-being |
| --- | --- | --- | --- | --- | --- |
| WE | 1 |  |  |  |  |
| Emotional labor | -0.414^***^ | 1 |  |  |  |
| Work support | 0.524^***^ | -0.364^***^ | 1 |  |  |
| Identifying value | 0.529^***^ | -0.383^***^ | 0.688^***^ | 1 |  |
| Caring about well-being | 0.521^***^ | -0.379^***^ | 0.707^***^ | 0.699^***^ | 1 |

Note: ^***^*p*<0.001.

**Abbreviations:** WE, work engagement; EL, Emotional labor.

# Table S2 Comparison of the average scores of each dimension item of different profiles of POS

| Dimensions | Class1 (low) | Class2 (medium) | Class3 (high) | *F* | Post hoc test |
| --- | --- | --- | --- | --- | --- |
|  | M (SD) | M (SD) | M (SD) |  |  |
| Work Support | 2.009 (0.565) | 3.094 (0.565) | 4.141 (0.565) | 1416.934^***^ | 3>2>1 |
| Identifying Value | 2.060 (0.587) | 3.138 (0.587) | 4.173 (0.587) | 1230.453^***^ | 3>2>1 |
| Caring about Well-being | 2.015 (0.539) | 3.084 (0.539) | 4.149 (0.539) | 1582.649^***^ | 3>2>1 |

**Note:** For all three dimensions of burnout, a higher score indicates a higher level of POS; ^***^*p*<0.001. Post hoc comparisons were performed using the Bonferroni correction.

**Abbreviations:** M, mean; SD, standard deviation.

# Table S3 Analysis of the interaction between the POS dimensions and emotional labor

| Variables | Unadjusted model *β* (95% CI) | *p* value | Adjusted model *β* (95% CI) | *p* value |
| --- | --- | --- | --- | --- |
| ***Work Support*** |  |  |  |  |
| Emotional labor | -0.349 (-0.407, -0.290) | <0.001 | -0.360 (-0.418, -0.301) | <0.001 |
| Work support | 1.486 (-1.480, 4.452) | 0.326 | 1.809 (-1.155, 4.772) | 0.231 |
| **Interaction: Emotional labor × Work support** | **0.103 (0.052, 0.154)** | **<0.001** | **0.105 (0.054, 0.156)** | **<0.001** |
| Constant | 60.654 | <0.001 | 60.517 | <0.001 |
| R² | 0.339 |  | 0.354 |  |
| Adjusted R² | 0.338 |  | 0.346 |  |
| ***Identifying Value*** |  |  |  |  |
| Emotional labor | -0.338 (-0.397, -0.279) | <0.001 | -0.348 (-0.407, -0.289) | <0.001 |
| Identifying value | -0.097 (-3.067, 2.874) | 0.949 | -0.042 (-3.006, 2.923) | 0.978 |
| **Interaction: Emotional labor × Identifying value** | **0.135 (0.083, 0.186)** | **<0.001** | **0.142 (0.090, 0.193)** | **<0.001** |
| Constant | 60.238 | <0.001 | 56.866 | <0.001 |
| R² | 0.344 |  | 0.36 |  |
| Adjusted R² | 0.342 |  | 0.353 |  |
| ***Caring About Well-being*** |  |  |  |  |
| Emotional labor | -0.341 (-0.400, -0.282) | <0.001 | -0.352 (-0.411, -0.293) | <0.001 |
| Caring about well-being | 0.400 (-2.589, 3.388) | 0.793 | 0.346 (-2.629, 3.322) | 0.819 |
| **Interaction: Emotional labor × Caring about well-being** | **0.123 (0.071, 0.174)** | **<0.001** | **0.133 (0.081, 0.185)** | **<0.001** |
| Constant | 60.33 | <0.001 | 63.876 | <0.001 |
| R² | 0.336 |  | 0.354 |  |
| Adjusted R² | 0.335 |  | 0.347 |  |

**Note:** Adjusted model controlled for gender,age, marital status, parental status, education, hospital level, hospital type, position, years working, professional title, employment type, weekly working hours, night shifts/month, and monthly income.

**Abbreviations:** POS, perceived organization support; SE, standard error; CI, confidence interval; LB, lower bound; UB, upper bound.

# Table S4 Analysis of the interaction between the total POS score and emotional labor

| Variables | Unadjusted model *β* (95% CI) | *p* value | Adjusted model *β* (95% CI) | *p* value |
| --- | --- | --- | --- | --- |
| Emotional labor (centered) | -0.73 (-0.94, -0.53) | <0.001 | -0.79 (-0.99, -0.58) | <0.001 |
| POS | 0.42 (0.38, 0.46) | <0.001 | 0.45 (0.41, 0.49) | <0.001 |
| Interaction: Emotional labor × POS^a^ | 0.0058 (0.0034, 0.0083) | <0.001 | 0.0063 (0.0039, 0.0088) | <0.001 |
| Constant | 9.20 (6.16, 12.25) | <0.001 | 5.16 (-4.11, 14.43) | 0.276 |
| R² | 0.387 |  | 0.420 |  |
| Adjusted R² | 0.386 |  | 0.405 |  |

**Note:** Adjusted model controlled for gender,age, marital status, parental status, education, hospital level, hospital type, position, years working, professional title, employment type, weekly working hours, night shifts/month, and monthly income.

^a^ Coefficients for continuous interaction terms are reported with additional decimal places to avoid loss of precision due to rounding.

**Abbreviations:** POS, perceived organization support; SE, standard error; CI, confidence interval; LB, lower bound; UB, upper bound.

# Table S5. Main and interaction effects of surface acting on work engagement across perceived organizational support profiles

| Variables | Main effect model  β (95% CI) | p value | Interaction model  β (95% CI) | p value |
| --- | --- | --- | --- | --- |
| Surface acting^a^ | -0.57 (-0.68, -0.47) | <0.001 | -0.88 (-1.14, -0.63) | <0.001 |
| POS (ref: Low) |  |  |  |  |
| Medium | 11.09 (9.05, 13.13) | <0.001 | 9.63 (7.30, 11.96) | <0.001 |
| High | 22.30 (19.91, 24.69) | <0.001 | 22.12 (19.50, 24.73) | <0.001 |
| Interaction: Surface acting × POS (ref: Low) |  |  |  |  |
| Surface acting × Medium | — | — | 0.19 (-0.10, 0.49) | 0.193 |
| Surface acting × High | — | — | 0.68 (0.36, 1.00) | <0.001 |
| Constant | 42.45 (32.28, 52.61) | <0.001 | 28.16 (18.65, 37.68) | <0.001 |
| R² | 0.334 |  | 0.345 |  |
| Adjusted R² | 0.317 |  | 0.327 |  |

**Note:** Both models were adjusted for sex, age, marital status, parental status, education, hospital level, hospital type, position, years of work, professional title, employment type, weekly working hours, monthly night shifts, and monthly income.

**Abbreviations:** POS, perceived organizational support; CI, confidence interval.

^a^ Surface acting was mean-centered in the interaction model.

# Table S6. Main and interaction effects of emotional display requirements on work engagement across perceived organizational support profiles

| Variables | Main effect model β  (95% CI) | p value | Interaction model β  (95% CI) | p value |
| --- | --- | --- | --- | --- |
| Emotional expression^a^ | -0.83 (-1.01, -0.65) | <0.001 | -1.24 (-1.68, -0.80) | <0.001 |
| POS (ref: Low) |  |  |  |  |
| Medium | 11.53 (9.47, 13.58) | <0.001 | 10.55 (8.27, 12.84) | <0.001 |
| High | 23.41 (21.03, 25.78) | <0.001 | 23.36 (20.80, 25.91) | <0.001 |
| Interaction: Emotional expression × POS (ref: Low) |  |  |  |  |
| Emotional expression × Medium | — | — | 0.20 (-0.30, 0.71) | 0.425 |
| Emotional expression × High | — | — | 0.96 (0.42, 1.49) | <0.001 |
| Constant | 38.39 (28.24, 48.54) | <0.001 | 26.67 (17.08, 36.26) | <0.001 |
| R² | 0.322 |  | 0.33 |  |
| Adjusted R² | 0.304 |  | 0.312 |  |

**Note:** Both models were adjusted for sex, age, marital status, parental status, education, hospital level, hospital type, position, years of work, professional title, employment type, weekly working hours, monthly night shifts, and monthly income.

**Abbreviations:** POS, perceived organizational support; CI, confidence interval.

^a^ Emotional display requirements were mean-centered in the interaction model.

# Table S7. Main and interaction effects of deep acting on work engagement across perceived organizational support profiles

| Variables | Main effect model β  (95% CI) | p value | Interaction model β  (95% CI) | p value |
| --- | --- | --- | --- | --- |
| Deep acting^a^ | -0.96 (-1.22, -0.70) | <0.001 | -1.82 (-2.57, -1.07) | <0.001 |
| POS (ref: Low) |  |  |  |  |
| Medium | 12.04 (9.97, 14.11) | <0.001 | 10.71 (8.37, 13.05) | <0.001 |
| High | 24.48 (22.11, 26.85) | <0.001 | 23.99 (21.40, 26.57) | <0.001 |
| Interaction: Deep acting × POS (ref: Low) |  |  |  |  |
| Deep acting × Medium | — | — | 0.50 (-0.33, 1.33) | 0.241 |
| Deep acting × High | — | — | 1.67 (0.80, 2.53) | <0.001 |
| Constant | 37.68 (27.14, 48.21) | <0.001 | 25.05 (15.37, 34.73) | <0.001 |
| R² | 0.307 |  | 0.318 |  |
| Adjusted R² | 0.289 |  | 0.299 |  |

**Note:** Both models were adjusted for sex, age, marital status, parental status, education, hospital level, hospital type, position, years of work, professional title, employment type, weekly working hours, monthly night shifts, and monthly income.

**Abbreviations:** POS, perceived organizational support; CI, confidence interval.

^a^ Deep acting was mean-centered in the interaction model.

# Table S8. Sensitivity analyses of the association between emotional labor and work engagement after additional adjustment for department fixed effects

| Variables | Main effect model β (95% CI) | p value | Interaction model β (95% CI) | p value |
| --- | --- | --- | --- | --- |
| Emotional labor^a^ | -0.35 (-0.41, -0.29) | <0.001 | -0.53 (-0.69, -0.38) | <0.001 |
| POS (ref: Low) |  |  |  |  |
| Medium | 10.72 (8.67, 12.78) | <0.001 | 9.15 (6.75, 11.54) | <0.001 |
| High | 21.85 (19.44, 24.25) | <0.001 | 21.73 (19.06, 24.41) | <0.001 |
| Interaction: EL × POS (ref: Low) |  |  |  |  |
| EL × Medium | — | — | 0.10 (-0.08, 0.27) | 0.269 |
| EL × High | — | — | 0.42 (0.23, 0.61) | <0.001 |
| Constant | 48.64 (38.01, 59.26) | <0.001 | 29.82 (20.07, 39.56) | <0.001 |
| R² | 0.344 |  | 0.356 |  |
| Adjusted R² | 0.322 |  | 0.334 |  |

**Note:** Both models were adjusted for sex, age, marital status, parental status, education, hospital level, hospital type, position, years of work, professional title, employment type, weekly working hours, monthly night shifts, monthly income, and department fixed effects. Coefficients for covariates and department indicators are not shown for brevity.

**Abbreviations:** EL, emotional labor; POS, perceived organizational support; CI, confidence interval.

^a^ Emotional labor was mean-centered in the interaction model.

# Table S9. Sensitivity analyses of the association between emotional labor and work engagement using department-clustered CR2 robust standard errors

| Variables | Main effect model β (95% CI) | p value | Interaction model β (95% CI) | p value |
| --- | --- | --- | --- | --- |
| Emotional labor^a^ | -0.35 (-0.54, -0.17) | 0.011 | -0.54 (-0.63, -0.45) | <0.001 |
| POS (ref: Low) |  |  |  |  |
| Medium | 10.65 (8.51, 12.79) | <0.001 | 9.01 (7.36, 10.66) | <0.001 |
| High | 21.74 (17.60, 25.88) | <0.001 | 21.58 (18.41, 24.74) | <0.001 |
| Interaction: EL × POS (ref: Low) |  |  |  |  |
| EL × Medium | — | — | 0.11 (-0.09, 0.30) | 0.278 |
| EL × High | — | — | 0.43 (0.07, 0.79) | 0.020 |
| Constant | 47.32 (33.84, 60.81) | <0.001 | 28.74 (22.38, 35.10) | <0.001 |
| R² | 0.34 |  | 0.353 |  |
| Adjusted R² | 0.323 |  | 0.335 |  |

**Note:** Both models were adjusted for sex, age, marital status, parental status, education, hospital level, hospital type, position, years of work, professional title, employment type, weekly working hours, monthly night shifts, and monthly income. Robust standard errors were estimated using department-clustered CR2 correction.

**Abbreviations:** EL, emotional labor; POS, perceived organizational support; CI, confidence interval; CR2, cluster-robust variance estimator with small-sample correction.

^a^ Emotional labor was mean-centered in the interaction model.
